# WIND (Workflow for pIRNAs aNd beyonD): a strategy for in-depth analysis of small RNA-seq data

**DOI:** 10.12688/f1000research.27868.2

**Published:** 2021-05-14

**Authors:** Konstantinos Geles, Domenico Palumbo, Assunta Sellitto, Giorgio Giurato, Eleonora Cianflone, Fabiola Marino, Daniele Torella, Valeria Mirici Cappa, Giovanni Nassa, Roberta Tarallo, Alessandro Weisz, Francesca Rizzo

**Affiliations:** 1 Laboratory of Molecular Medicine and Genomics, Department of Medicine, Surgery and Dentistry ‘Scuola Medica Salernitana’, University of Salerno, Baronissi, Salerno (SA), 84081, Italy; 2 Genomix4Life, via S. Allende 43/L, Baronissi, Salerno (SA), 84081, Italy; 3 Clinical Research and Innovation, Clinica Montevergine S.p.A., Mercogliano, Mercogliano, 83013, Italy; 4 CRGS (Genome Research Center for Health), University of Salerno Campus of Medicine, Baronissi, Salerno (SA), 84081, Italy; 5 Department of Medical and Surgical Sciences, Magna Graecia University, Viale Europa, Catanzaro, 88100, Italy; 6 Department of Experimental and Clinical Medicine, Molecular and Cellular Cardiology, Magna Graecia University, Viale Europa, Catanzaro, 88100, Italy

**Keywords:** piRNA, small RNA sequencing, ncRNA-expression, workflow

## Abstract

Current bioinformatics workflows for PIWI-interacting RNA (piRNA) analysis focus primarily on germline-derived piRNAs and piRNA-clusters. Frequently, they suffer from outdated piRNA databases, questionable quantification methods, and lack of reproducibility. Often, pipelines specific to miRNA analysis are used for the piRNA research *in silico*. Furthermore, the absence of a well-established database for piRNA annotation, as for miRNA, leads to uniformity issues between studies and generates confusion for data analysts and biologists.

For these reasons, we have developed WIND ( **W**orkflow for p **I**RNAs a **N**d beyon **D**), a bioinformatics workflow that addresses the crucial issue of piRNA annotation, thereby allowing a reliable analysis of small RNA sequencing data for the identification of piRNAs and other small non-coding RNAs (sncRNAs) that in the past have been incorrectly classified as piRNAs. WIND allows the creation of a comprehensive annotation track of sncRNAs combining information available in RNAcentral, with piRNA sequences from piRNABank, the first database dedicated to piRNA annotation. WIND was built with Docker containers for reproducibility and integrates widely used bioinformatics tools for sequence alignment and quantification. In addition, it includes Bioconductor packages for exploratory data and differential expression analysis. Moreover, WIND implements a "dual" approach for the evaluation of sncRNAs expression level quantifying the aligned reads to the annotated genome and carrying out an alignment-free transcript quantification using reads mapped to the transcriptome. Therefore, a broader range of piRNAs can be annotated, improving their quantification and easing the subsequent downstream analysis. WIND performance has been tested with several small RNA-seq datasets, demonstrating how our approach can be a useful and comprehensive resource to analyse piRNAs and other classes of sncRNAs.

## Introduction

Advances in the field of Next-Generation Sequencing and big data analysis have led to the identification of several small non-coding RNA (sncRNA) classes, some of which are still poorly characterised ^1, 2^. Among others, the most investigated include microRNAs (miRNAs), small interfering RNAs (siRNAs), PIWI-interacting RNAs (piRNAs), small nuclear (snRNAs) and small nucleolar RNAs (snoRNAs). Increasing evidence demonstrates that the different sncRNAs constitute interconnected networks of molecules with key-regulatory functions in multiple biological processes, including physiological events, organism development or even disease ^3^.

piRNAs represent an heterogeneous group, ubiquitous in most animal's germline cells, with lack of conserved sequences and few common structural features in the various species, due to the highly adaptive nature of the piRNA pathway ^4^. Germline piRNAs typically have a 21–35 nt length, a strong bias for 5'-end uridine signature and a 2'-O-methyl group at their 3'-end ^5^. Most of them are transcribed by either mono-directional or bidirectional genomic clusters, specific regions ranging from <1 kb to >100 kb, giving rise to a long, single-stranded precursor and further processed in multiple mature piRNAs through enzymatic cleavage. A subset of piRNAs present an adenosine bias at position 10, a feature indicating their biogenesis through the ping-pong cycle, a mechanism by which the cleavage of the target RNA is coupled with the production of a second population of target-specific piRNAs. They interact with PIWI proteins of the Argonaute (AGO) family, forming a silencing complex able to suppress transposable elements, regulate target’s gene expression at both epigenetic and post-transcriptional level and defend from viral infections ^6^. These piRNA functions are well studied in the animal germline, however in somatic cells, their role needs to be further elucidated. Additional studies have revealed that piRNA dysregulation can contribute to the onset of several diseases ^7^. Notably in cancer, the abnormal expression of piRNAs has been associated with tumour initiation, progression, and metastasis formation and these molecules have shown the potential to be useful diagnostic tools and therapeutic targets as well as biomarkers for cancer prognosis ^8^.

A limitation in understanding their function and use in clinical practice is the lack of a comprehensive and reliable method for their identification in tissues others than germline. A common strategy for piRNAs identification is based on mapping the reads obtained from high-throughput small RNA libraries to the genome and then annotate to small RNA databases for quantification. Most of the piRNA sequences identified so far have been deposited in databases such as piRNABank ^9^, piRNAdb ( https://www.pirnadb.org/), piRNAclusterDB ^10^ and others. However, data collected in these repositories mainly include germline piRNAs, while somatic piRNAs represent a minor fraction. In addition, piRNAs in somatic tissues and human cancers are less abundant than in germline, thereby leading to a more difficult identification and characterisation. Although piRNAs were initially confounded with fragments of longer RNAs, functional piRNAs have been identified to derive from fragments of rRNAs, tRNAs, snoRNAs, and post-transcriptionally processed mRNA ^11– 13^. Another level of complexity is represented by their genomic origin(s) and their actual amount, since identical sequences of piRNA can be produced by multiple genomic loci, resulting in very low precision and sensitivity.

For all the reasons stated above, and since existing workflows and tools for piRNA-analysis, usually, focus on the identification and quantification of piRNA clusters (PILFER ^14^, unitas ^15^) or use outdated piRNA databases, we decided to implement a useful workflow for small RNA sequencing data analysis, able to analyse all classes of sncRNAs but especially designed for piRNA identification. We created a workflow that provides a quick method to integrate different piRNA databases in one annotation track, a two-method approach for small RNA identification, annotation and quantification, and an output with several ready-to-publish plots and statistics. Additionally, we packaged the entire workflow in several Docker ^16^ containers avoiding the annoying problems related to the installation and libraries dependencies. Finally, we applied it to different small RNA datasets, highlighting that piRNAs are dysregulated in breast cancer tissues and may play an important role in maintaining the stemness of MCF7 spheroid-enriched cancer stem cells (CSCs).

## Methods

In this study, we implemented a workflow for small RNA sequencing data analysis, defined WIND ( **W**orkflow for p **I**RNAs a **N**d beyon **D**) ^17^ **,** designed for a comprehensive identification and quantification of small-RNAs and especially of piRNAs. We deployed it exploiting the Docker containerisation approach, allowing us to integrate multiple bioinformatics tools. In detail, we created two Docker images where we adopted broadly used tools for pre-processing, reads alignment, identification and quantification of sncRNAs, and all downstream analyses. We also integrated the already available container made for Salmon ^18^ for transcriptome analysis. This solution takes into account best practices for reproducibility, versatility and ease of use, as the software deployment is fast and efficient. It can be used in various operating systems with only the requirements of the Docker engine and some minimum adjustments for processing power and RAM for the most memory demanding tools.

### Workflow

**Figure 1.**
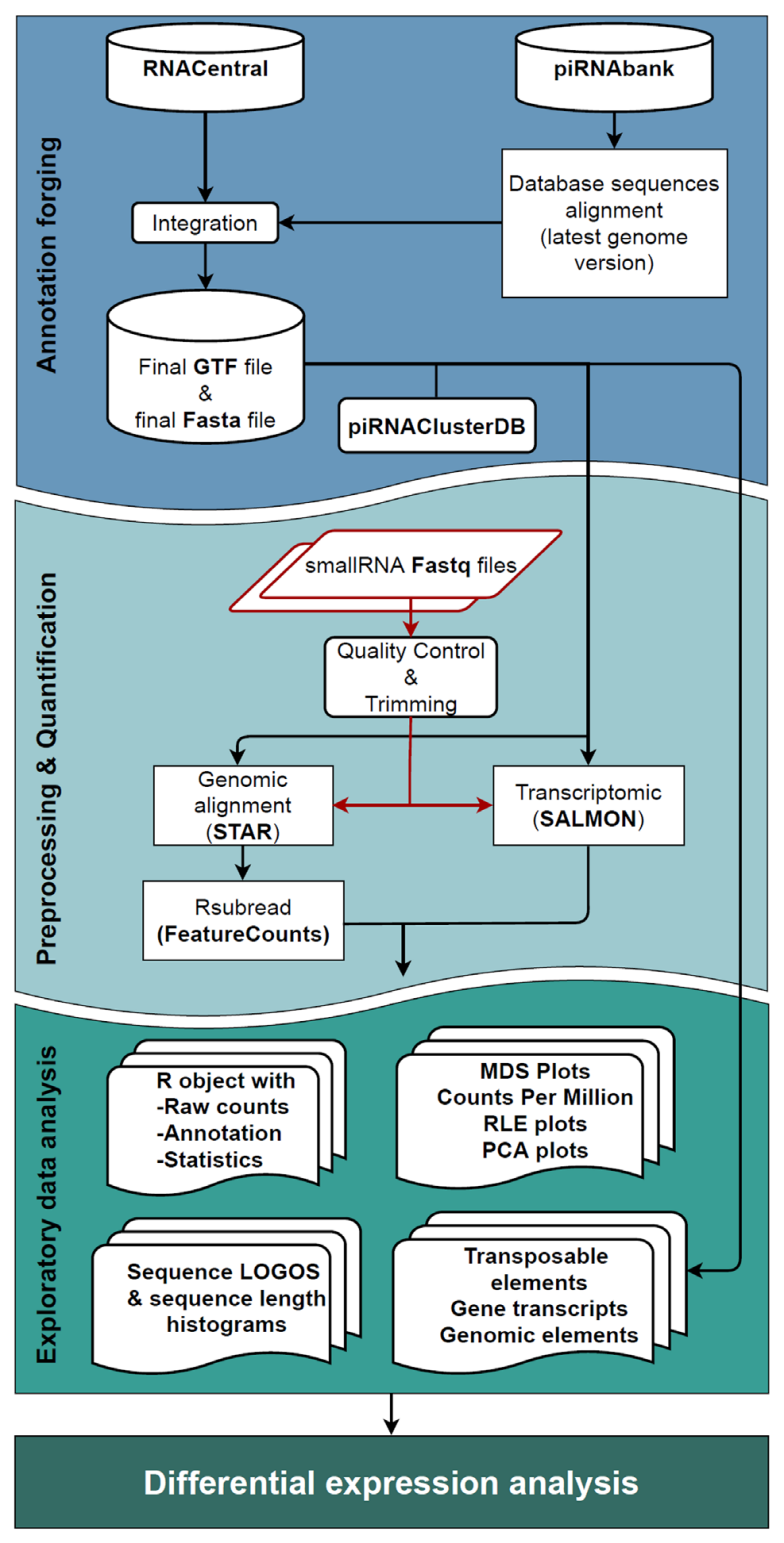
Workflow schematic representation. The *Annotation forging* step, represented in blue, is the creation of a GTF file, where the two input databases (piRNABank and RNAcentral) are merged to produce the new small RNA annotation track, that together with the Fasta files constitute the inputs of the following step. In *Pre-processing & Quantification* step (light blue area), the user's fastq files undergo through the quality check, and the adapter removal followed by the two quantification approaches (completed by Salmon, and STAR with FeatureCounts software) that perform in parallel alignment and the quantification of reads. In the green box, representing the *Exploratory data analysis* phase, are displayed all the possible results produced by the workflow. The data analyst could also pursue differential expression analysis if that is the desirable outcome.

The workflow consists of three significant steps ( Figure 1):

1. *Annotation forging:* the generation of the annotation files for small RNA sequences used in the next quantification step.
2. *Pre-processing and quantification:* pre-processing, alignment and quantification of the reads assigned to sncRNAs (using a dual approach: genomic and transcriptomic analysis).
3. *Exploratory data analysis:* result exploration of both quantification methods in parallel and Differential Expression (DE) with two different methodologies (edgeR ^19, 20^ and limma-voom ^21^).

***Annotation forging.*** The first step of WIND, the *Annotation forging* (blue box in Figure 1) is the creation of the annotation track. In this step, we tried to reduce and potentially correct the issues regarding piRNA annotation, such as the presence of multi-mapped piRNA IDs, the inconsistencies among piRNA databases, and the misleading annotation of small RNA fragments. In particular, 667,944 human and 1,399,813 mouse sequences were acquired from piRNABank (02-May-2007, Version 1, hg19). Duplicates and multi-mapped sequences were collapsed, leading to 23,439 and 39,986 unique sequences for human and mouse, respectively. Afterwards, these unique sequences were realigned to the latest version of the reference genome (Gencode ^22^ primary assembly, GRCh38.p13 for human, and GRCm38.p6 for mouse) using STAR aligner ^23^ with the following parameters: --alignIntronMax 0, --outFilterMultimapNmax 100, --outFilterMatchNmin 16, and --outFilterMismatchNmax 0. Further on, 446,265 human and 180,780 mouse small RNA sequences from RNAcentral ^24^ (v16, 28/09/2020) were utilised to complete the database (for details see *Extended data:* Supplementary Table 1). Then, the sequences from both databases were filtered with respect to their length, keeping only those with less than 100 bases in length, since our primary interests are piRNAs and sncRNAs, and keeping only those that correspond to standard chromosomes. Moreover, a re-classification of the piRNABank sequences was made. According to Tosar *et al*. ^25^, a small percentage of annotated piRNAs are probably piRNA-sized fragments of sncRNAs (rRNAs, tRNAs, YRNAs, snRNAs, and snoRNAs) or intermediates of miRNA biogenesis and potentially act as contaminants in the quantification step of the workflow. For this reason, piRNABank sequences matching sequences from RNAcentral with different sncRNA types (biotype) other than piRNA are re-categorised with the biotype from RNAcentral.

Subsequently, as it is well established that mature piRNAs have a length of around 21~35 bases ^26^, before the final assembly of the sequences from both databases, the piRNABank sequences shorter than 69 bases (<69bp) are integrated with RNACentral sequences. Furthermore, we excluded the piRNA genomic ranges falling in regions annotated as protein coding, exons or CDS from the GENCODE annotation file. However, the user can choose to skip this filtering and obtain a “less stringent” GTF file. Moreover, inspired by Tosar *et al.* 2021 ^27^, we included, as metadata, those piRNAs sequences that are inside other sncRNAs and lncRNAs from GENCODE annotation file. The obtained sequences are finally exported to Fasta and GTF (Gene Transfer Format) file format. Eventually, we decided to provide the information available on piRNACLusterDB ^10^ as metadata in the final GTF file.

These tracks, for human and mouse species, have been included in the GitHub repository of the workflow and are available for users ( https://github.com/ConYel/wind); therefore, the annotation forging step can be skipped. The workflow can also be used for any other species, but in this case, it would be necessary for the user to run the *Annotation forging* step with the specific genome and small RNA sequences, respectively.

The mapping of these piRNA sequences to the genome has revealed that the piRNAs can derive from two types of genomic locations: discrete genomic loci (the piRNA clusters) and protein-coding genes (e.g. UTRs, introns) ^28– 30^. Using bumphunter ^31^ package in the workflow, we were able to obtain piRNA origin information and provide it as the first additional file of the annotation. Since piRNAs are involved in the maintenance of genome stability through the silencing of transposable elements ^32^, in this step, we also report a GTF file with the intersection between the genomic positions of small RNAs, the various categories of Transposable Elements (TEs) and information about the TE class, family and gene. Briefly, the GTF file is created with the related Fasta file; then the Genomic_Region_info, Multimapping_piRNA_info and Transposable_Elements_info files are reported which carry information about the genomic topology for the sequences in the GTF file. Likewise, these files are available in the GitHub repository (GRCh38 and GRCm38), for future usage by the data analyst.

***Pre-processing and quantification.*** The second step of the workflow, *Pre-processing and quantification* (light blue box in Figure 1), consists of a quality control check of the small RNA-seq data, carried out using the FastQC tool ^33^, followed by the adapter removal using Cutadapt ^34^ and by another quality control check using FastQC again. After these initial steps, the workflow exploits two different approaches for the quantification of sncRNAs. In particular, one uses alignment to a reference genome with STAR and then quantification of aligned reads with FeatureCounts ^35^; the other one uses Salmon (an aligner-free method) for the estimation of transcript-level abundance. We named the two approaches “genomic” and “transcriptomic” based on how the two methods work. Both approaches have positive and negative features. Undoubtedly, with STAR, the reads are aligned on a reference genome, Salmon instead is an alignment-free quantification method, able to prioritise the association of a feature with a specific site on a transcriptome. On the one hand, STAR could associate a read on multiple sites creating a complete list of identified regions, but this makes it more difficult to determine the genomic locations of origin, thus requiring more computational work. On the other hand, Salmon is a transcriptome quantifier able to correct for fragment GC-content and positional bias, which improves the accuracy of abundance estimates and potentially the sensitivity of subsequent DE analysis.

To ensure the proper alignment of sncRNA reads to the genome, we used the following options for STAR aligner (as used in SPAR workflow ^36^): --alignIntronMax 1, --outFilterMultimapNmax 100, --outFilterMismatchNmax 1 and --outFilterMatchNmin 14. For Salmon, to be suitable for small RNA reads, the following options were applied: --seqBias, --gcBias, --numBootstraps 100, and --validateMappings as was suggested from the work of Wu *et al.* ^37^. Resulting files, from the previous step, are imported to R using the Bioconductor packages: tximport (for Salmon) and Rsubread (for FeatureCounts), as DGEList objects (edgeR). After reads count, a FeatureCounts object is reported as an R object (.RDS) for an easy and fast way to import it in R. Moreover, we decided to record the assigned reads from both Salmon and FeatureCounts as BAM files.

***Exploratory data analysis.*** The last step of the workflow is the *Exploratory data analysis* (EDA), which includes the filtering of low expressed small RNAs, the normalisation procedures performed in parallel for both FeatureCounts and Salmon, and then the visualisation of the results according to the suggested EDA workflows ^38– 41^ from Bioconductor ^42^. Finally, the workflow provides several useful output files: text and RDS files with filtered or normalised reads, information about the filtering step, Multidimensional Scaling (MDS) plots, biodetection plots, expression per small RNA category plot (countsbio), distance-matrix plot, hierarchical clustering plots with various normalisation methods, Principal Components Analysis (PCA) plots, Relative Log Expression (RLE) plots ^43, 44^, voom-derived plots, sequence length barplots, and piRNA sequence logos. Briefly, for each dataset analysed, 9 RDS files, 17 tab-delimited files with all the statistics from alignment, filtering and annotation plus the filtered and normalised reads in counts per million (CPM), and 24 PDF files with several exploratory data analysis plots for each of the two methods used are generated. Furthermore, we also provided a script for the creation of ping-pong and strand coverage plots exploiting ssviz ^45^ and ggbio ^46^ R packages from Bioconductor.

Eventually, the gene expression data can be further compared using the DE analysis module, which allows calculating logarithmic fold change values using limma-voom or edgeR methods, and finally, both results (Salmon and FeatureCounts) can be merged and visualised together using heatmaps ^47^. Then, the data analyst can choose to use the union of the results, and either consider all the molecules identified by at least one of the two methods, or use the intersection of the results and consider only the molecules supported by two methods. The differentially expressed molecules can be further used for piRNA target prediction analysis (included in the code) which was inspired by the similar module of iSMART tool ^48^.

This workflow is structured to provide maximum flexibility to the user, who can modify several elements. In each step, alterations can be made regarding the tools or the databases used according to the needs of the data analyst, while the workflow strategy remains the same. Specifically, in the first step, the GTF file can be enriched with more sequences of interest or a completely new GTF file could be created for any species. Currently, the first step has been performed on human and mouse sncRNA sequences for the generation of the GTF files (included in the GitHub repository), but the same approach could be utilised for any well-annotated genome that has enough small RNA sequences reported. In the second step, it is possible to use different tools for quality control, adapter trimming, aligning of the reads, e.g. Subread ^49^ or HISAT2 ^50^ or a different "alignment-free" RNA-seq quantification method, as Kallisto ^51^.

### Operation

The workflow was run on CentOS Linux release 7.8.2003 (Core) with Docker Engine - Community v19.03.13 and in R v4.0.0, with Bioconductor v3.12.

### Validation and datasets

The complete workflow has been tested on several datasets to evaluate whether this worked in the identification of known piRNAs, low abundant molecules and in different species. Specifically, we have evaluated the performance of the transcriptomic approach on sncRNA identification, and particularly on piRNAs, for which this method has been tested here for the first time. We created a small dataset where spike-in sequences of piRNA-like molecules were added to the input RNA. For this purpose, RNA of metastatic colon cancer cell line (COLO 205), where piRNA's population has been already characterised ^52^, was used. To mimic the behaviour of true piRNAs, a synthetic set of 4 piRNA-like molecules was used, including two non-methylated (SS-22 and SS-28) and two methylated (mSS-22 and mSS-28) of different lengths (22 nt and 28 nt). Spike-ins were chemically synthesised at Exiqon, adapting the sequences described in Locati *et al.* ^53^ to our conditions and the pool of 4 molecules was used at three different concentrations, with a final amount of 0.3 × 10^9 (dil_A), 0.3 × 10^10 (dil_B) and 0.3 × 10^11 (dil_C) molecules/ug of RNA. Small RNA libraries were prepared using 1 μg of total RNA with a TruSeq small RNA Sample Prep Kit (Illumina, San Diego, Canada) and sequenced on the NextSeq 500 platform (Illumina, San Diego, CA, USA) as previously described in Sellitto *et al.* 2019 ^52^ (samples are available on ArrayExpress, Accession number E-MTAB-9772: COLO205_Dil_A, COLO205_Dil_B, COLO205_Dil_C). Furthermore, we also exploited the samples processed with sodium periodate/β-elimination (samples are available on ArrayExpress, Accession number E-MTAB-8115: Treated_COLO205_1, Treated_COLO205_2, Treated_COLO205_3, Treated_testis_1) as an additional control for the quantification algorithms. Indeed, sodium periodate oxidation strongly reduces the non-methylated molecules allowing to see a drastic change in non-methylated spike-ins concentration.

To test the performance of WIND, in both high-piRNA and low-piRNA expression conditions, we used Human Testis RNAs (BioChain Institute Inc, Newark, CA, USA) and COLO 205 cell line RNAs (samples are available on ArrayExpress. Accession number E-MTAB-8115: Non_treated_Testis_1 and Non_treated_COLO205_1, Non_treated_COLO205_2, Non_treated_COLO205_3, Treated_COLO205_1, Treated_COLO205_2, Treated_COLO205_3, Treated_testis_1; Accession number E-MTAB-9782: Non_treated_Testis_2 and Non_treated_Testis_3). To test the workflow on mouse data, we used two samples of mouse adult Cardiac Myocyte (samples are available on ArrayExpress, Accession number E-MTAB-9866: aCM1, aCM2) ^54, 55^.

Furthermore, we also exploited two public datasets to test our workflow thoroughly including the differential expression module dataset, consisting of two experimental conditions in triplicates, MCF-7 enriched CSCs spheroids and monolayer cultures (Accession number GSE68246 ^56, 57^); and a subset of 18 samples from TCGA-BRCA ^58, 59^, using 9 Primary Solid Tumour versus 9 Solid Tissue Normal corresponding samples.

## Results

The goal of this study was to create a robust workflow for the identification and quantification of piRNA sequences in small RNA sequencing data. It focuses on elucidating and solving one of the most challenging issues of this kind of analysis, the annotation controversies of piRNAs, thus providing relatively accurate detection of the piRNA expression patterns. As a first point, a unique GTF file was generated for human and mouse species, starting from sequences obtained from the two widely used databases (piRNABank and RNAcentral) for piRNAs and sncRNAs, respectively. The GTF file was created as described in the *Methods* obtaining 149,549 different genomic locations corresponding to 39,812 sequences in human and 925,759 distinct genomic locations corresponding to 95,205 sequences in mouse for all small RNA types (see *Extended data:* Supplementary Table 1). Furthermore, in humans, from the 39,812 sequences coding for small RNAs, 28,000 were classified as piRNA, and 19,203 of them were found in common between RNAcentral and piRNABank; instead, 8,444 were found only in RNAcentral and 353 only in piRNABank. Additionally, in the mouse genome, from 95,205 sequences of small RNAs, 65,632 were categorised as piRNA, 34,306 were in common between the two databases and on the contrary, 29,114 were unique to RNACentral, and 2,213 were exclusive to piRNABank.

To test WIND thoroughly, we used several datasets with different characteristics: data produced in house, data available in a public repository, samples which include internal controls (spike-ins), datasets from different species (human and mouse), a dataset including different experimental conditions, and a dataset of tumour tissues (for more details see *Methods* and *Data availability*). First, we compared the quantification capability of the two methods implemented in the workflow. In particular, we evaluated the performance of the transcriptomic approach on piRNA quantification, as this method to our knowledge, has not yet been used to analyse this sncRNA class. For this reason, we decided to apply the workflow on an own-made dataset, in which spike-in sequences of four piRNA-like molecules, two non-methylated and two methylated at three different concentrations, were included (see *Methods* for details). Exploiting this feature, we were able to assess the high efficiency of both (genomic and transcriptomic) approaches in quantifying the spike-ins, as demonstrated by the very similar results obtained by the different methods. Supplementary Table 2 ( *Extended data*) summarises the results obtained for the 4 piRNA-like molecules calculated using three methods: iSMART, FeatureCounts, and Salmon. The results show that all approaches can identify and quantify all the types of piRNA-like sequences (methylated, non-methylated, treated and not treated, and of different length) correctly.

**Figure 2.**
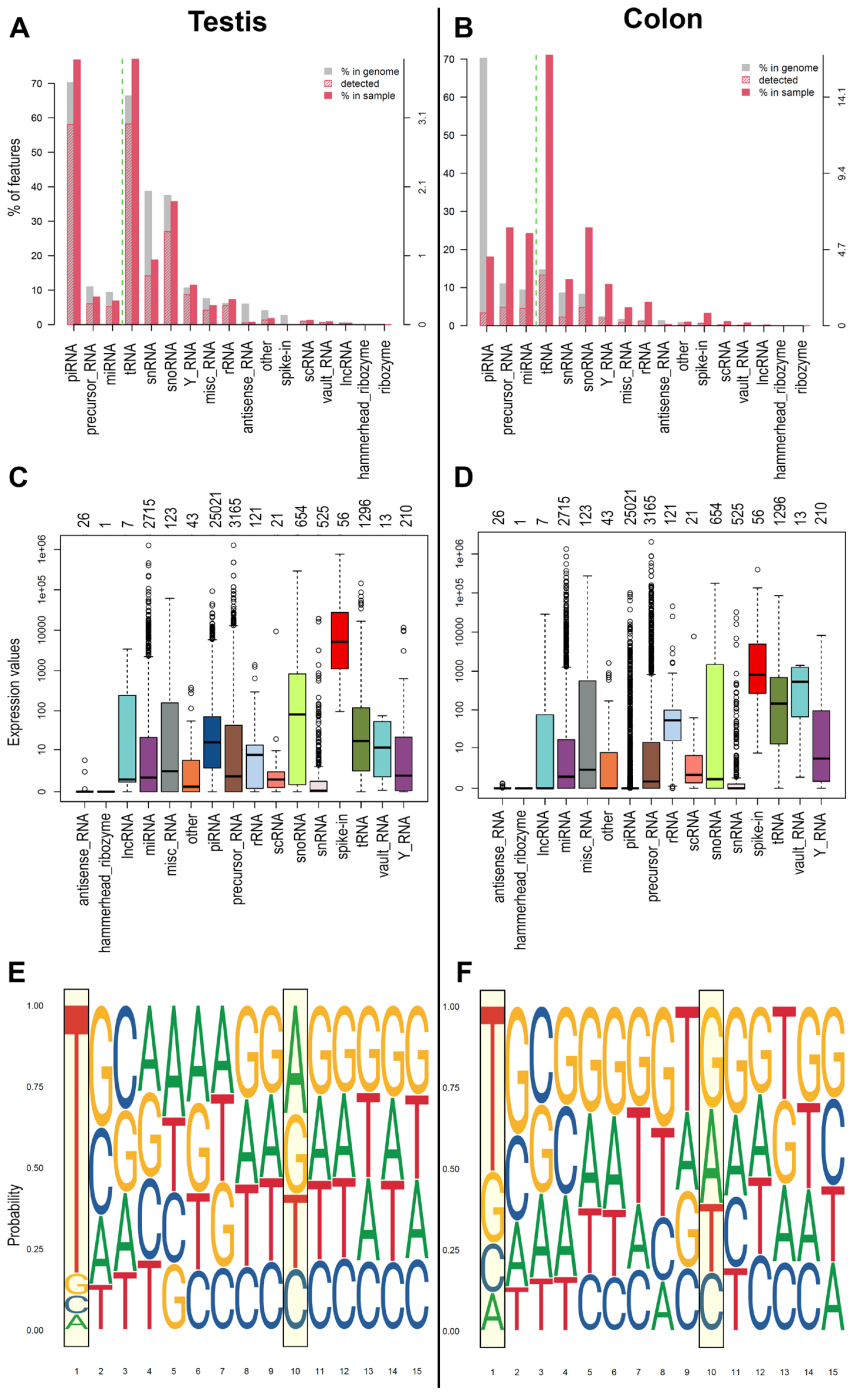
Example of plots generated by WIND. **A**) and **B**) Biodetection plots (genomic approach) from NOIseq reporting: percentages of each sncRNA type called "biotype" on the genome (grey bar) for one of the samples; the proportion detected in each sample (red stripes bar); the percentage of each biotype within the sample (red bar). The biotypes on the right side of the green dashed line are the least abundant, and the reference values are reported on the Y right axis. **C**) and **D**) CountsBio plots (genomic approach) from NOIseq showing the count distribution for each biotype displayed as boxplots. Numbers on top of the plot show how many sncRNAs are detected per biotype in the entire dataset analysed. Different colours indicate different sncRNA classes. **E**) and **F**) Sequence Logo (1-15 bps) extracted from the piRNA sequence of the expressed piRNAs found in each group of samples (transcriptomic approach). **A**, **C**, **E** represents the results obtained for one representative testis samples, while **B**, **D**, **F** represent one representative COLO 205 sample.

For a long time, piRNAs have been considered exclusively expressed in germline cells, but recently, it has been reported by several studies their presence also in somatic and pathologic tissues ^5, 52, 60– 62^. Germinal cells generally show the most significant number and a higher level of expression of piRNAs. Starting from this knowledge, we tested the workflow on small RNA data obtained from human testis samples and tumour cell line (COLO205) to assess the capability to detect piRNAs in high and low concentration. Using WIND, we analysed the dataset and represented the results as plots of biodetection ( Figure 2A and B) and countsBio ( Figure 2C and D) per sample from NOISeq ^63^ package. Biodetection plots are made from raw data in order to explore: a) the percentages of each small RNA type (named "biotypes") in the genome (referred to the whole set of small RNAs provided); b) the proportion detected in each sample; c) the percentage of each biotype within the sample. The countsBio plots, instead, show the count distribution for each biotype displayed as box plots, and the number of sncRNAs detected per biotype. Here, the two biodetection plots show, as expected, the presence of higher percentage of piRNA in testis sample respect to the COLO 205 cells (~75% in testis and ~20% in COLO 205; Figure 2A and B). Moreover, considering the countsBio plots ( Figure 2C and D), it is also possible to assess piRNAs higher median expression in testis if compared to the COLO 205 cells. Finally, we also produced sequence logos for the expressed piRNAs in the two sample types. These plots indicate if the bias for uridine at first position base or the adenine at the 10 ^th^ position of the sequence exists and if there are other biases in the 15 first bases of the sequences ^64^. As expected, both groups showed a strong bias for uridine at the first position (drawn as thymine in the plots), in accordance with the preferential binding of PIWI proteins to transcripts starting with U. A bias, albeit with a much weaker signal, was also evident toward adenine at position 10 in testis group, a hallmark of piRNAs generated by the ping-pong cycle and typical feature of germinal cells ( Figure 2E and F).

For this analysis, we applied a stringent approach; thus, we considered as expressed only those molecules that were identified by both methods (genomic and transcriptomic). Then, we found that 7324 piRNAs were identified in testis and 223 in COLO 205 cells. Therefore, this workflow was able to efficiently identify a good number of piRNAs in somatic cells, where low levels of expression make the procedure more complicated, even when very stringent analysis parameters are used.

It is worth mentioning that, as detailed in the *Methods*, this workflow operates using two methods in parallel, each of which is able to identify sncRNAs with different performance. Applying the two algorithms together (considering the union of the results) allows the identification of an enriched number of molecules. The final user can decide, based on specific interests, which results should take into consideration, the union of the two approaches, only one, or the intersection.

We also evaluated the performance of the workflow for piRNA identification in the mouse. Specifically, we analysed small RNA-seq samples from mouse adult cardiac myocyte (aCM). In these samples, we were able to identify, considering the union of the genomic and transcriptomic approach, ∼290 different piRNAs per samples (see *Extended data:* Supplementary Table 3 for the details of the two analysis in comparison). We found that the piRNA population identified in aCM represents 12% of all reads assigned to small RNAs, and the top 100 expressed molecules are listed in Supplementary Table 3 ( *Extended data*).

**Figure 3.**
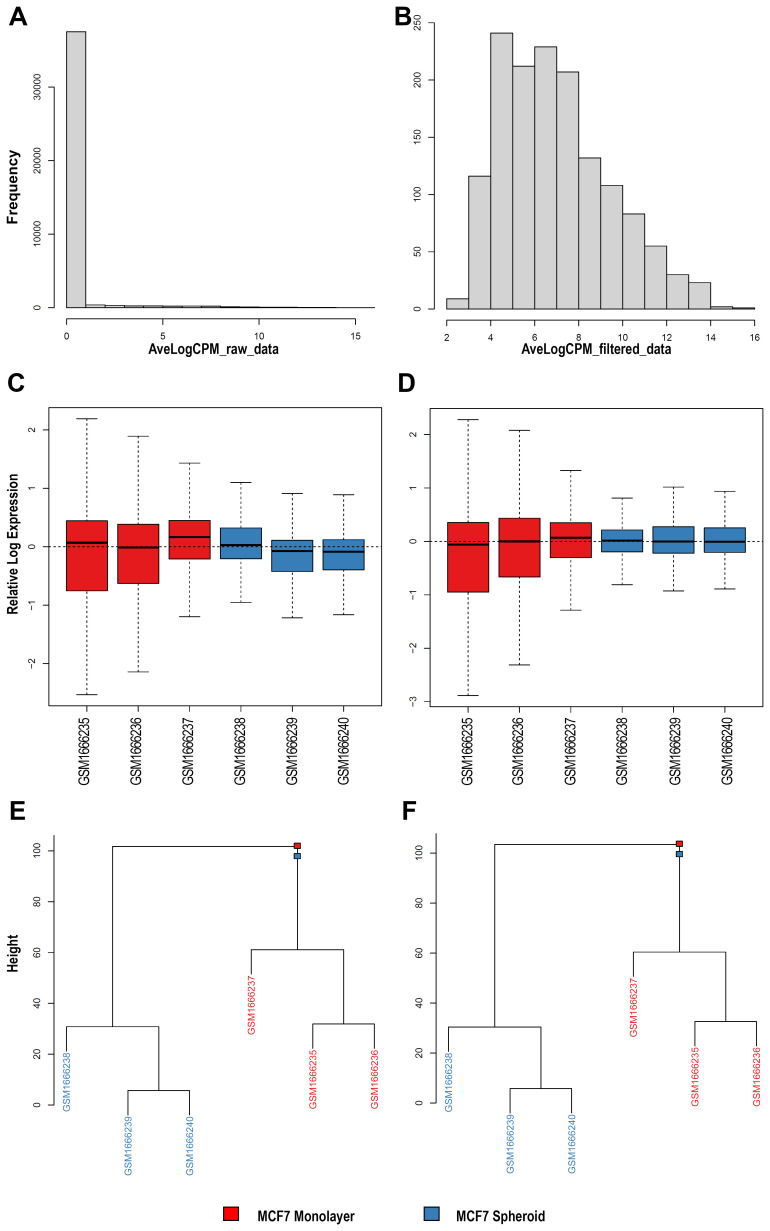
Exploratory data analysis plots generated by WIND. **A**– **B**) Histograms of average log _2_ Counts Per Million (CPM) among all samples before ( **A**) and after ( **B**) filtering with one of the selected methods (EdgeR filtering in this case) for sncRNA data. **C**– **D**) Relative Log Expression (RLE) plots for each normalisation method, made with the use of plotRLE function for all the sncRNA data. As an example, only the first two plots (with TMM ( **D**) and without normalisation ( **C**) for the filtered counts derived from FeatureCounts) are shown. **E**– **F**) Hierarchical Clustering plots, exploiting all the sequenced sncRNA data, with multiple clustering methods and different normalisation methods. As an example, only the first two plots (with TMM ( **D**) and without normalisation ( **C**) for the filtered counts derived from FeatureCounts). In black and brown are shown the two different groups (monolayer and spheroid).

**Figure 4.**
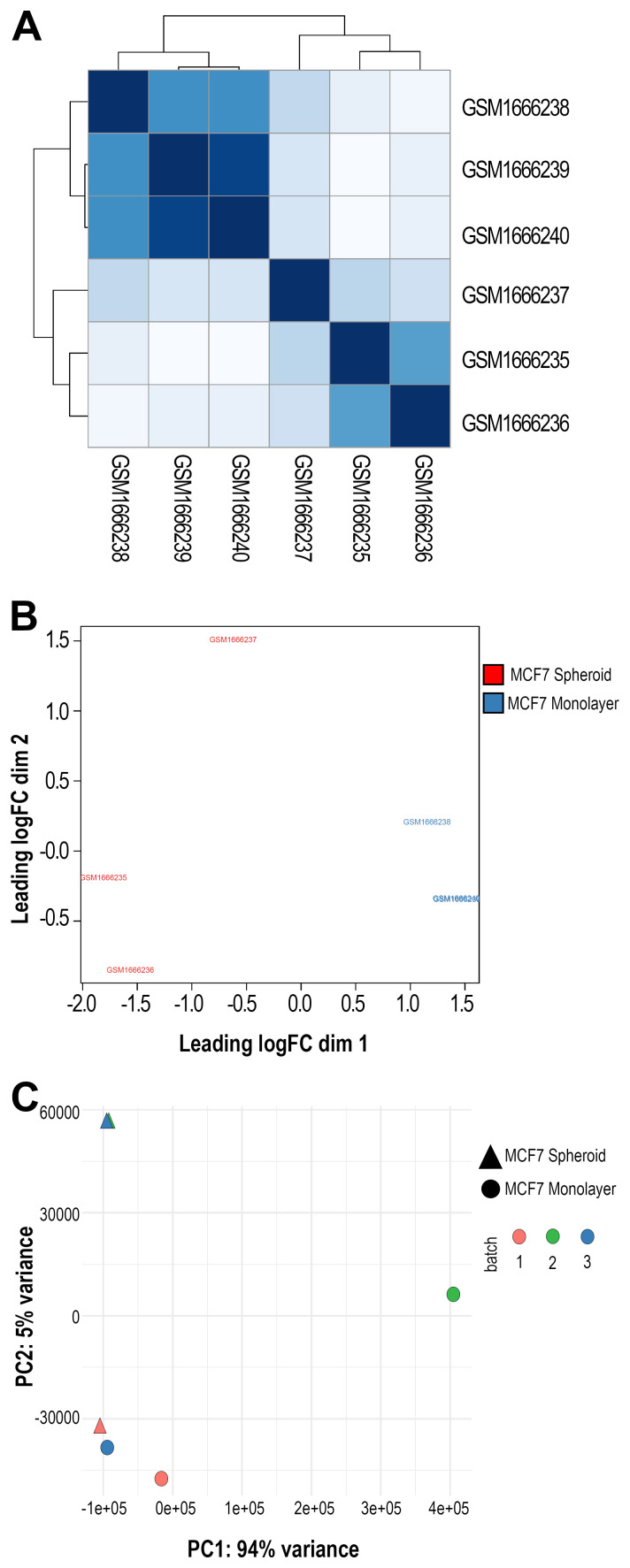
Sample group clustering plots. **A**) Correlation plot showing samples' distances in GSE68246 dataset. The darker the colour, the more correlated they are. **B**) Multidimensional Scaling (MDS) plot using all the sequenced data and one of the normalisation methods applied in the workflow (in this case, TMM) made with plotMDS() function from EdgeR. In black and brown are shown the two different groups (monolayer and spheroid). **C**) Principal Components Analysis (PCA) plot displaying the first two Principal Components using all the sncRNA molecules data. Each sample is shown with different colours (depending on the group) and different symbols (depending on the batch).

**Figure 5.**
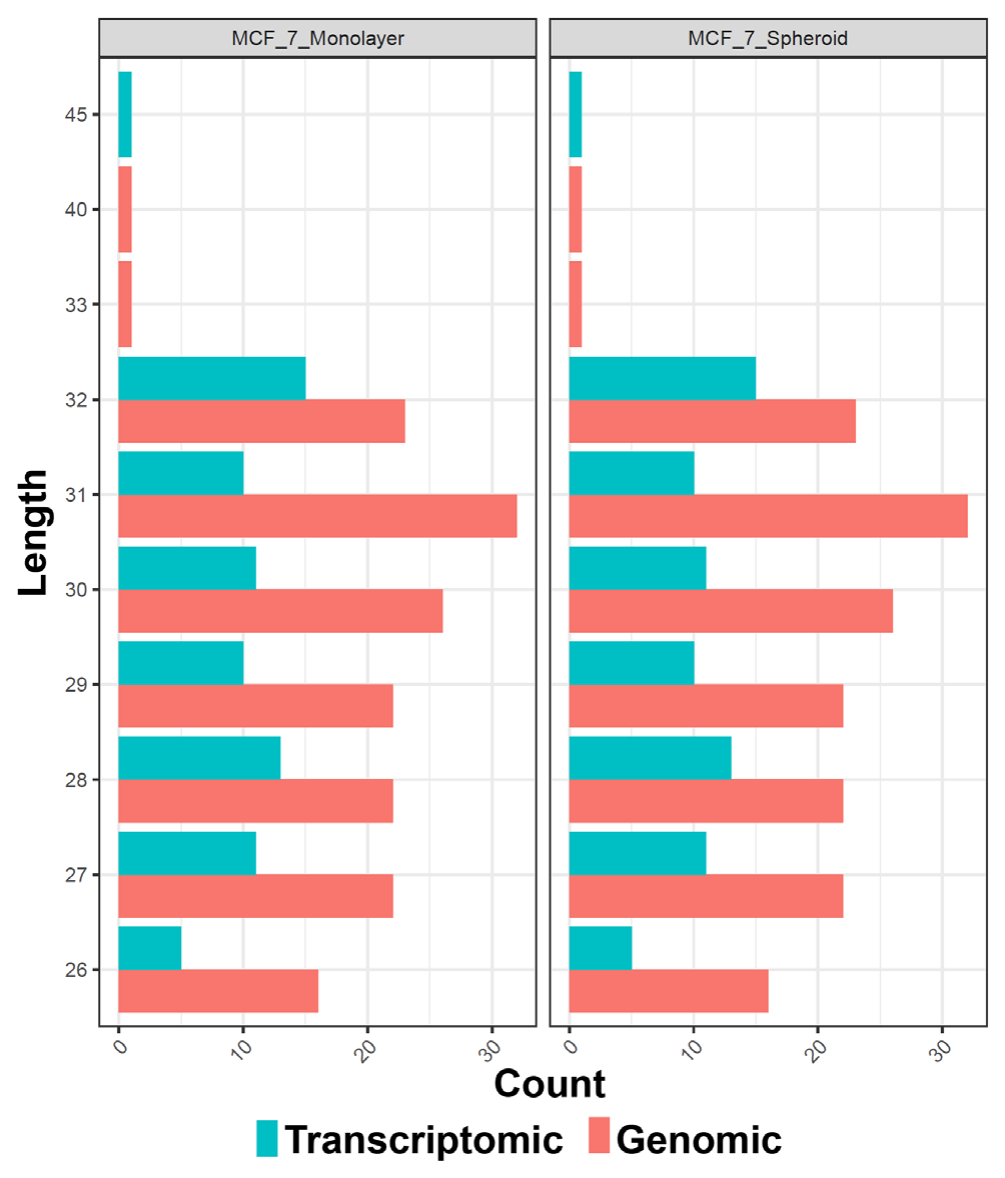
Barplots of the length of piRNA classes with respect to each experimental group (in this case monolayer and spheroid MCF7). The colours indicate the two different methods of quantification (genomic and transcriptomic).

Moreover, to test the accuracy of the workflow across diverse sets of data, we moved to a public dataset. Recent findings have indicated that the role of piRNAs may not be only limited to germ cells, but may be extended to the regulation of cancer, promoting a stem-like state of tumour cells ^65^. Therefore, we selected a dataset (GSE68246) to compare the piRNA profiles of breast spheroid-enriched CSCs against parental MCF7 cells and also generated in this case files, statistics and plots with WIND that are all available on the GitHub repository. On the expression data, filtering for low-expressed features was first carried out, then two of the NOIseq filters (1 count per million, and proportional filtering) or the EdgeR were applied, filtering by group with and without the specific batch. The resulting objects were reported as RDS files and, for all the analysed sequences, a histogram ( Figure 3A and B) with the average log _2_ CPM before and after filtering of the counts was made using the edgeR filtering. Finally, the normalisation of all the counts was carried out with multiple methods: TMM ^66^, TMMwsp (TMM with singleton pairing), RLE ^67^, limma-Voom, with and without quality weights quantile ^68^, Voom, with and without quality weights using the TMM normalisation, and Voom with and without quality weights exploiting the TMMwsp normalisation. To visualise the unforeseen sources of variation and to control whether the normalisation applied was correct, RLE plots ( Figure 3C and D) were generated for all the sequenced data, for each normalisation method and for the not normalised, filtered data. An RDS object was also exported with the list of all normalised objects, and hierarchical clustering ( Figure 3E and F) was then performed on previous data with various normalised methods. We applied the Euclidean distance and the methods of Ward's, complete and average linkage. Furthermore, a correlation plot ( Figure 4A) with sample-to-sample distances was made to show the similarities and dissimilarities between samples on all sncRNA data. In order to check for batch effects and get the summarised effects of the experimental categories, MDS plots ( Figure 4B) and the first two Principal Components on a PCA plot were reported ( Figure 4C). From the GTF file, sequences' lengths were extracted and combined with information about the expressed molecules to draw the barplots ( Figure 5), allowing to underline the differences between the two methods or between the groups of interest. Alongside, in this case, the sequences' logos for only the expressed piRNAs were generated. Moreover, we reported a tab-delimited file with the mean CPM per biological group, as it is useful to know these values for further studying or visualisation. All the files, plots and statistics are available in the GitHub repository.

**Figure 6.**
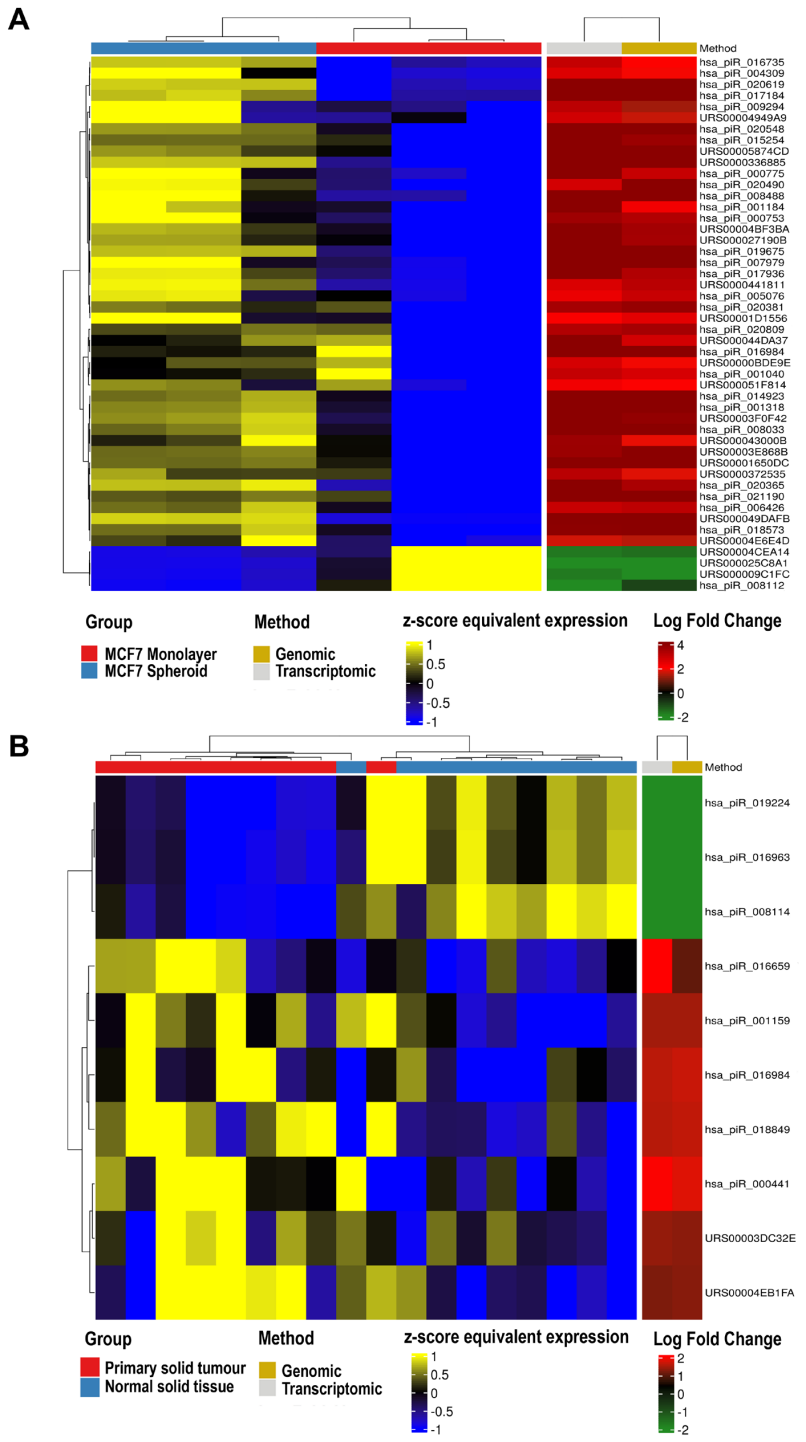
Differential expression analysis. **A**) Heatmap of differentially expressed piRNAs in 3 MCF7 Spheroid samples versus 3 MCF7 Monolayer (GSE68246 public dataset) found in common with both approaches (genomic and transcriptomic). **B**) Heatmap of differentially expressed piRNAs among 9 Primary Solid Tumour versus 9 Solid Tissue Normal from TCGA found in common with both approaches (genomic and transcriptomic).

Ultimately, we performed the differential expression analysis on the results of both methods (genomic and transcriptomic), and the union of the comparisons was reported ( *Extended data:* Supplementary Table 4). Our workflow identified 466 differentially expressed sncRNAs (p-value ≤ 0.05) using both methods and 352, considering the adjusted p-value ≤ 0.05. 63 miRNAs were found DE, in common with Boo *et al.* ^56^. Most importantly, we were able to identify 181 expressed piRNAs, 48 of which differentially expressed (adj. p-value < 0.05) between spheroids and parental cells, with 44 of them up- and 4 down-regulated ( Figure 6A). Their log-fold changes were varying from -2.60 to 8.05, and 20 of them derive from the sequences found in RNAcentral while 28 from piRNABank, thus showing the importance of including both databases in the final GTF file. Of these 48 DE piRNAs, three of them (DQ570940, DQ571550, DQ578783) have also been found DE in the work of Vella *et al.* ^69^ in cardiosphere-derived cells. This suggests a possible functional role of this group of piRNAs in the stemness of cancer cells, independently from the tissue type.

As a final test, we exploited sncRNA data of 18 samples from TCGA-BRCA, 9 Primary Solid Tumor versus 9 Solid Tissue Normal ( *Extended data:* Supplementary Table 5). We identified 10 piRNAs DE out of 235 DE sncRNA molecules with both approaches (genomic and transcriptomic). In the heatmap ( Figure 6B), it is possible to note that the two approaches obtained equivalent results, and the clustering approach showed a good clustering between tumour and normal samples. Interestingly, some of the identified piRNAs have been previously described as related to cancer progression in tissues like kidney and lung (DQ581033 ^70^, DQ593398 - DQ592932 ^71^). In addition, from the 235 DE sncRNA, 64 are reported as miRNA and most importantly, we found the cancer-specific MIR-8 (now reported as mir-141 and mir-200) upregulated as previously reported by Hoadley *et al.* ^59^. In order to acquire possible functional information about the DE piRNAs, we predicted their possible target RNAs (using the code included in the workflow), and we identified 11 protein-coding genes ( *Extended data:* Supplementary Table 5). Most of them were predicted to bind their targets at the 3' UTR and 4 at the 5' UTR. The functional enrichment analysis of the 11 predicted piRNA targets, using the EnrichR online tool ^72^, revealed that they might be involved in regulating "signal transduction that contributes to a DNA damage checkpoint" (GO:0072422), a biological process that has a vital role in cancer progression.

## Conclusions

In this paper, we describe a novel bioinformatics workflow, WIND ^17^, for the identification and analysis of piRNA from small RNA sequencing data. The main innovations of WIND are: a Docker containerisation approach for the complete analysis, the integration of two databases for piRNA annotation, a dual-method for detection and quantification of piRNAs (named as "genomic" and "transcriptomic" in this article), and the creation of ready-to-use plots and statistics for the interpretation of the results. The idea was born in order to cope with the absence of a gold-standard pipeline for piRNA identification and annotation. We tried to solve many issues related to small RNA sequencing data analysis and, in particular, piRNA identification and quantification. For this reason, the first step was to deploy multiple Docker containers set up to run all the steps of the workflow without installing tools, software or libraries. After this, we focused on the creation of an easy method to integrate data from distinct databases (RNACentral and piRNABank). As described in methods, we were able to assess the deep diversity between the databases. Indeed, it was possible to notice not only differences in numbers of piRNAs annotated between the two databases (both in human and mouse genome) but also inconsistencies in the annotation or in the classification (e.g. the same molecule is classified as piRNA in one and as miRNA in the other). Actually, combining databases usually produces discrepancies and working with sncRNA sequences that have multiple annotations is troublesome. However, with this step, it is possible to obtain a unique GTF file that merges this information (all ids and genomic locations associated with that specific molecule) that can be used for piRNA identification and annotation. The main part of our workflow consists of two detection methods for piRNAs described above as "genomic" and "transcriptomic". For the genomic part, we decided to perform an alignment using STAR. STAR is a well-known genomic aligner that uses a reference genome to compute read alignments. For the transcriptomic part, we used Salmon to produce accurate transcript-level quantification estimates from sncRNA sequencing data. Salmon's main innovation is the use of quasi-mapping (accurate and very fast-to-compute read alignments). However, even if the transcriptomic approach proved to be working well, it has been demonstrated that for the identification of some sncRNAs might not be as efficient as the genomic approach ^37^. For this reason, we set the methods to improve sncRNAs identification, following the suggestions of previous works ^36, 37^. Our idea was to combine the two approaches in order to evaluate the similarities between the results obtained and then ameliorate the identification of piRNAs. The last step was to create a Differential Expression module and, most importantly, the automatic creation of plots and statistics useful for the interpretation of data and results.

To test WIND, we applied it to several sncRNA datasets. Working on the first dataset, were we used the spike-in approach, we found a good consistency between the different methods in the detection of piRNA-like molecules, highlighting the efficiency of both approaches in piRNA quantification. Furthermore, the test on germline and somatic tissues revealed that the two methods, even when a stringent filter is applied, are able to assess the presence of piRNAs also in tissues where they are not abundant. In addition, the workflow is also functional in different species, as shown by the results obtained on the mouse genome. Finally, we also tested WIND on two published datasets, comprising tumour cell lines and tissues. Our workflow, also in this instance, was able to identify efficiently piRNAs and find differentially expressed molecules (not previously investigated) and to recognise, in general, a significant number of sncRNAs.

In conclusion, WIND is a complete dockerised workflow, usable by bioinformaticians and data analysts who want to explore small RNA sequencing data globally, but specifically designed and optimised for piRNAs. WIND allows going from raw data to plots and statistics ready for publication thanks to fast and efficient software implementation, making it very useful in the field of small RNA research.

## Data availability

### Underlying data

ArrayExpress: Monitor the efficiency of "WIND: A Workflow for pIRNAs aNd beyonD" for the identification of single-stranded (SS) spike-in piRNA-like molecules in smallRNA-seq, Accession number E-MTAB-9772: https://www.ebi.ac.uk/arrayexpress/experiments/E-MTAB-9772/

ArrayExpress: Monitor the efficiency of "WIND: A Workflow for pIRNAs aNd beyonD" for the identification of piRNA molecules in small RNA-seq, Accession number E-MTAB-9782: https://www.ebi.ac.uk/arrayexpress/experiments/E-MTAB-9782/

ArrayExpress: Monitor the efficiency of "WIND: A Workflow for pIRNAs aNd beyonD" for the identification of piRNA in mouse samples, Accession number E-MTAB-9866: https://www.ebi.ac.uk/arrayexpress/experiments/E-MTAB-9866/

ArrayExpress: Analysis of the 3’-end of piRNAs in the COLO 205 cell line through sodium periodate (NaIO4) /β-Elimination treatment and small RNA-Seq, Accession number E-MTAB-8115: https://www.ebi.ac.uk/arrayexpress/experiments/E-MTAB-8115/

NCBI Gene Expression Omnibus: miRNA transcriptome profiling of spheroid-enriched cells with cancer stem cell properties in human breast MCF-7 cell line, Accession number GSE68246: https://www.ncbi.nlm.nih.gov/geo/query/acc.cgi?acc=GSE68246

Selected samples from the Genomic Data Commons Data Portal ^73^ have been accessed and analysed from the TCGA-BRCA project: https://portal.gdc.cancer.gov/projects/TCGA-BRCA

### Extended data

Zenodo: Supplementary tables, http://doi.org/10.5281/zenodo.4730400 ^74^.

This project contains the following extended data:

- **Supplementary Table 1**. Statistics of GFT files obtained for human and mouse genome. The file reports the data of the filtering process and the final GTF data.
- **Supplementary Table 2**. Spike-in quantification. For each sample are shown the percentage of each piRNA-like molecules, respect to the raw reads count, using three quantification methods.
- **Supplementary Table 3**. Statistics of sncRNA data analysis for mouse cardiomyocytes. The file reports the results obtained using the two methods applied in the workflow and the list of top 100 expressed piRNAs.
- **Supplementary Table 4**. Differentially Expressed molecules found for GSE68246 dataset. In yellow are highlighted miRNA DE in common with Boo *et al.* ^56^, in red and green are highlighted the up- and down-expressed molecules respectively, in light blue and cyan the molecules with a p-value and adjusted p-value less than 0.05 respectively.
- **Supplementary Table 5**. Differentially Expressed molecules found for BRCA TCGA dataset. In red and green are highlighted the up- and down-expressed molecules respectively, in light blue and cyan the molecules with a p-value and adjusted p-value less than 0.05 respectively. For DE piRNA molecules, the predicted possible target RNAs are also provided.

Data are available under the terms of the Creative Commons Attribution 4.0 International license (CC-BY 4.0).

## Software availability

Workflow available from: https://github.com/ConYel/wind

Archived workflow as at time of publication: http://doi.org/10.5281/zenodo.4289908 ^17^.

License: MIT

All software packages used throughout this workflow are publicly available through the Bioconductor project ( http://bioconductor.org), or the Comprehensive R Archive Network ( https://cran.r-project.org) and all bioinformatics tools are freely available as Docker containers on https://hub.docker.com/r/congelos/.
