# Supplementary material for: WIND (Workflow for pIRNAs aNd beyonD): a strategy for in-depth analysis of small RNA-seq data
Source: F1000Res. 2021 Jul 12;10:1. Originally published 2021 Jan 4. [Version 3] doi: 10.12688/f1000research.27868.3 (PMC8276195; doi:10.12688/f1000research.27868.3)
Supplement: Peer review history

# Peer review: Reviewer response for version 3

**DOI:** 10.5256/f1000research.58313.r89408

**Published:** 2021-07-12

**Authors:** Juan Pablo Tosar

**Affiliations:** 1 Analytical Biochemistry Unit, School of Science, Universidad de la República, Montevideo, Uruguay; 2 Functional Genomics Unit, Institut Pasteur de Montevideo, Montevideo, Uruguay

I am satisfied with the author responses and with version 3 of this paper.

Is the rationale for developing the new method (or application) clearly explained?

Yes

Is the description of the method technically sound?

Yes

Are the conclusions about the method and its performance adequately supported by the findings presented in the article?

Partly

If any results are presented, are all the source data underlying the results available to ensure full reproducibility?

Yes

Are sufficient details provided to allow replication of the method development and its use by others?

Yes

Reviewer Expertise:

Molecular and cell biology

I confirm that I have read this submission and believe that I have an appropriate level of expertise to confirm that it is of an acceptable scientific standard.

# Peer review: Reviewer response for version 2

**DOI:** 10.5256/f1000research.56589.r85365

**Published:** 2021-06-08

**Authors:** Wen Yao

**Affiliations:** 1 National Key Laboratory of Wheat and Maize Crop Science, Henan Agricultural University, Zhengzhou, China

The authors have addressed all my concerns. I have no further comments.

Is the rationale for developing the new method (or application) clearly explained?

Yes

Is the description of the method technically sound?

Yes

Are the conclusions about the method and its performance adequately supported by the findings presented in the article?

Yes

If any results are presented, are all the source data underlying the results available to ensure full reproducibility?

Yes

Are sufficient details provided to allow replication of the method development and its use by others?

Yes

Reviewer Expertise:

Genomics, Bioinformatics, Genetics.

I confirm that I have read this submission and believe that I have an appropriate level of expertise to confirm that it is of an acceptable scientific standard.

# Peer review: Reviewer response for version 2

**DOI:** 10.5256/f1000research.56589.r85364

**Published:** 2021-05-21

**Authors:** Juan Pablo Tosar

**Affiliations:** 1 Analytical Biochemistry Unit, School of Science, Universidad de la República, Montevideo, Uruguay; 2 Functional Genomics Unit, Institut Pasteur de Montevideo, Montevideo, Uruguay

The authors have made important efforts to address my concerns raised in the previous version of this manuscript. 

 This is a method article. As such, I think the method is designed in a thoughtful manner and tested with relevant controls, so it could constitute a useful resource to other researchers interested in piRNA annotation.

 However, although the abstract and discussion are method-oriented, the result section is strongly supportive of widespread somatic piRNA expression in mammals, which is controversial. I give the authors the credit to address this relevant research question by removing as much confounders from their GTF files as possible, and for their willingness to redo most of their analysis after my initial concerns. But an analysis of the sequence of many of the top 100 expressed piRNAs in mouse cardiomyocytes (Supplementary Table 3) shows that they still have 100% identity to rRNAs (e.g., mmu_piR_039147, mmu_piR_005109), snoRNAs (mmu_piR_032974, mmu_piR_034793, mmu_piR_025007), etc. 

 Thus, although the design of the workflow intends to remove all piRNAs with overlap to mRNAs or ncRNA fragments, there seems to be a problem in the filtering step, and some of the article's claims are not fullfilled. 

 My intention is not to further delay acceptance of this manuscript. But the fact that most remaining piRNAs are still non-coding RNA fragments should be at least analyzed and acknowledged. Supplementary tables should contain a list of the most abundant piRNAs in all the cases analyzed (including COLO 205 cells, etc), and sequences should be provided. Ideally, the authors could try to understand why many piRNAs with an overlap to ncRNA fragments are not being removed from their GTF files, fix the problem and redo the analysis. 

*Minor:*

 mSS28 seems to be resistant to periodate treatment, but mSS22 is not. Is my interpretation correct (Supp Table S2)

Is the rationale for developing the new method (or application) clearly explained?

Yes

Is the description of the method technically sound?

Yes

Are the conclusions about the method and its performance adequately supported by the findings presented in the article?

Partly

If any results are presented, are all the source data underlying the results available to ensure full reproducibility?

Yes

Are sufficient details provided to allow replication of the method development and its use by others?

Yes

Reviewer Expertise:

Molecular and cell biology

I confirm that I have read this submission and believe that I have an appropriate level of expertise to confirm that it is of an acceptable scientific standard, however I have significant reservations, as outlined above.

# Response

**Published:** 2021-06-30

**Authors:** Francesca Rizzo

**Affiliations:** University of Salerno, Italy

Q1. This is a method article. As such, I think the method is designed in a thoughtful manner and tested with relevant controls, so it could constitute a useful resource to other researchers interested in piRNA annotation.

 However, although the abstract and discussion are method-oriented, the result section is strongly supportive of widespread somatic piRNA expression in mammals, which is controversial. 

**A1. The aim of this article was to develop a workflow for sncRNA identification, focused on piRNAs, with the intention of detecting piRNAs (and not only them) independently of the tissue of origin. Indeed, we tested the WIND performance in detecting the presence of piRNAs in both somatic and germline tissues, but demonstrating the somatic expression of piRNAs is beyond this article’s aim. We used these types of data as proof of the sensing power of our workflow in identifying piRNAs even in the presence of low expressed molecules.**

 Q2. I give the authors the credit to address this relevant research question by removing as much confounders from their GTF files as possible, and for their willingness to redo most of their analysis after my initial concerns. But an analysis of the sequence of many of the top 100 expressed piRNAs in mouse cardiomyocytes (Supplementary Table 3) shows that they still have 100% identity to rRNAs (e.g., mmu_piR_039147, mmu_piR_005109), snoRNAs (mmu_piR_032974, mmu_piR_034793, mmu_piR_025007), etc. Thus, although the design of the workflow intends to remove all piRNAs with overlap to mRNAs or ncRNA fragments, there seems to be a problem in the filtering step, and some of the article's claims are not fullfilled. My intention is not to further delay acceptance of this manuscript. But the fact that most remaining piRNAs are still non-coding RNA fragments should be at least analyzed and acknowledged.

**A2. The results obtained show that the workflow can detect piRNA sequences that have been annotated in various Databases as “piRNAs” until now. Although precautions have been taken for the removal of as many as possible sequences that could potentially not be piRNAs, in specific cases the removal of the sequences using only bioinformatic data is not possible, but would require a further study, possible only through wet-lab experiments (using various techniques), which can clarify the true nature of the molecule. As the workflow considers what in the databases exist ab initio, it is not possible to arbitrarily remove all piRNA sequences that have a high similarity or are included in the sequence of other sncRNAs, without the risk of eliminating real piRNAs from the analysis.**

**Referring to the reviewer’s example, using BLASTN [Zheng Zhang, Scott Schwartz, Lukas Wagner, and Webb Miller (2000), "A greedy algorithm for aligning DNA sequences", J Comput Biol 2000; 7(1-2):203-14] on the sequence of “mmu_piR_039147”, we get as a result :**

**“ Mus musculus large subunit ribosomal RNA gene, partial sequence” with Max. score: 38.2, Total Score: 38.2, Query Cover: 90%, E value:0.073, Per. Ident: 100.00%.**

**The sequence of mmu_piR_039147 is: CCTCGACACAAGGGTTTGTAA and the sequence that is found to have a partial match with it is CCTCGACACAAGGGTTTGT, which is 2 nucleotides less.**

**The genomic ranges of mmu_piR_039147 in the GTF file are**

**chr6 87997228-87997248 -**

**chr9 118674951-118674971 -**

**In the GTF file (gencode.vM25.primary_assembly.annotation.gtf.gz) that we have used to search if these ranges are included in other sncRNAs, there are no overlaps with any sncRNAs for mmu_piR_039147. As we have seen from BLASTN, a part of the sequence is identical to “ Mus musculus large subunit ribosomal RNA gene, partial sequence” but that doesn’t mean that it is from the same genomic location and is, therefore, an rRNA fragment. Of course, this is just one case and other piRNA sequences match 100% with a part of another sncRNA, but they can also come from a completely different genomic region.**

**We agree with the reviewer that some molecules can be fragments or other sncRNAs incorrectly annotated. However, it is not possible at present, without wet-lab experiments, to distinguish fragmentation products from molecules that can come from other ncRNAs such as rRNAs, tRNAs, and snoRNAs, as recently proposed by Barreñada *et al.* 2020 (“Diversification of piRNAs expressed in PGCs and somatic cells during embryonic gonadal development”, Barreñada *et al.* 2020).**

**We believe that WIND workflow must show all the possible reproducible results and all the useful information and then the data analyst, with the help of the biologist and eventually experimental approaches, will be able to deepen the study and possibly focus on the dubious sequences, to establish whether they are or no piRNA when ambiguity exists. As we already know that these issues exist, we have included as a metadata column on the GTF file, for all the piRNA genomic regions that fall inside other sncRNAs, this information, thus the data analyst can directly check if what identified as expressed or differentially expressed has multiple annotations.**

**In other words, for example, if the piRNA identified by the workflow is inside a miRNA precursor or tRNA, this information can be found in the metadata column “GENCODE_annot” of the GTF. Moreover, it is possible to obtain a file with the detail of all mapped reads assigned to a specific piRNA and verify if they are multi-mapped or not. The data analyst can use this file to see if most of the reads are associated with a complete sequence of a piRNA or if some (for example shorter reads) also map to another sncRNA.**

**In conclusion, the workflow tries to deal with the issues of piRNA annotation that the databases have until now; however, in our opinion, to completely solve these issues, it is not conceivable to use only an in-silico approach but it is necessary to combine it with wet laboratory techniques, as immunoprecipitation (IP) of PIWI-containing ribonucleoprotein complexes and/or the RNA periodate treatment, to define which sequence is truly a piRNA. Our contribution, with this workflow, is to mitigate the already existing problems and to use it as a backbone for potential future strategies for sncRNA analysis.**

**In order to be clear about these concerns and to clarify to the users the type of results provided by WIND, we added these sentences in the conclusion section:**

***“It is important to clarify that WIND deals with issues and discrepancies present in the original piRNA databases and it recategorizes piRNAs using sequences and genomic ranges information. However, it is still possible to find overlap between piRNAs and other ncRNAs, due to sequence similarity, ab initio categorization of a sequence as piRNA without further wet-lab validation, or real sncRNAs derived piRNAs. However, this information is stored in the final GTF. The purpose is to give complete information to allow studying piRNAs and other sncRNAs, highlighting possible ambiguities of the in silico analysis, which researchers will be able to evaluate and eventually resolve with wet-lab experiments, such as immunoprecipitation (IP) of PIWI-containing ribonucleoprotein complexes and/or the RNA periodate treatment”*.**

 Q3. Supplementary tables should contain a list of the most abundant piRNAs in all the cases analyzed (including COLO 205 cells, etc), and sequences should be provided. Ideally, the authors could try to understand why many piRNAs with an overlap to ncRNA fragments are not being removed from their GTF files, fix the problem and redo the analysis.

**A3. All resulting files (the top 100 expressed piRNAs, along with other 28 info files and 21 PDF files for each analyzed dataset) are published in the GitHub repository and can be found in this https://github.com/ConYel/wind/tree/main/Datasets_analysis folder. All the information about the sequences of each sncRNA is included in the GTF files.**

**Furthermore, during the 2nd revision, our main point was to remove any incongruencies between piRNA genomic positions that fall inside gene coding regions. The existence of piRNAs with an overlap to sncRNA fragments is a well-known issue that our workflow tried to mitigate, however, despite our best efforts, it is not possible to completely remove all these molecules without further wet-lab investigation. For now, we prefer to keep the sequences that are inside sncRNAs and give that information to the users as metadata. In this way, the researchers have all the information and can decide if, for the molecules of interest, it is necessary to confirm the molecule's nature with the help of wet-lab experiments. Moreover, the workflow is under constant development and better ways of solving this kind of issue could be found in the future and integrated with the workflow. Any data analyst or biologist that has a specific request, about the workflow, is able to make a suggestion or even improve it by himself.**

 Q4.Minor:

 mSS28 seems to be resistant to periodate treatment, but mSS22 is not. Is my interpretation correct (Supp Table S2)

**A4. Thanks to the reviewer, we noticed a missing part of the table in the submitted Supplementary table 2. We have fixed the error and we re-uploaded it.**

# Peer review: Reviewer response for version 1

**DOI:** 10.5256/f1000research.30818.r80430

**Published:** 2021-03-25

**Authors:** Wen Yao

**Affiliations:** 1 National Key Laboratory of Wheat and Maize Crop Science, Henan Agricultural University, Zhengzhou, China

The authors developed a new computational pipeline for analysis of piRNAs in small RNA sequencing data. The methods were elaborated clearly in the manuscript. The pipeline was tested with real small RNA sequencing data of human and mouse. The manuscript is well written and the WIND pipeline will be a good tool facilitating the analysis of piRNAs. I have several comments and suggestions for the authors.

**Major:**

1. The title should be revised as *“a strategy for in-depth analysis of small RNA-seq data”* is confusing. The WIND pipeline mainly focuses on the analysis of piRNA, which is only a category of all small RNA species. For example, miRNA is ignored by WIND.
2. I agree with the author that the key to piRNA analysis is the comprehensive and accurate identification of piRNAs and piRNA precursor. However, only piRNABank and RNAcentral were used in the “Annotation forging” step of WIND while updated databases of piRNA and piRNA cluster had been published. I suggest the authors integrating piRBase ^1^ and piRNA cluster database ^2^ in the WIND pipeline.
3. Figure 1. *“small RNA Fastq files”* were not used in the *“Annotation forging”* step, and should be placed in the *“Pre-processing and quantification”* step.
4. In the *“Annotation forging”* step, I noticed two length thresholds (100 bp and 69 bp) were used when building the *“final Fasta file”*. So why 100 and 69?

**Minor:**

1. Figure 5 is bar plot rather than histogram.
2. Figure 6B - part of the legend at the bottom is obscured.

Is the rationale for developing the new method (or application) clearly explained?

Yes

Is the description of the method technically sound?

Yes

Are the conclusions about the method and its performance adequately supported by the findings presented in the article?

Yes

If any results are presented, are all the source data underlying the results available to ensure full reproducibility?

Yes

Are sufficient details provided to allow replication of the method development and its use by others?

Yes

Reviewer Expertise:

Genomics, Bioinformatics, Genetics.

I confirm that I have read this submission and believe that I have an appropriate level of expertise to confirm that it is of an acceptable scientific standard, however I have significant reservations, as outlined above.

## References

[1] : piRBase: a comprehensive database of piRNA sequences. *Nucleic Acids Research* .2019;47(D1) : 10.1093/nar/gky1043 D175-D180 10.1093/nar/gky1043 PMC632395930371818

[2] : piRNA cluster database: a web resource for piRNA producing loci. *Nucleic Acids Res* .2016;44(D1) : 10.1093/nar/gkv1265 D223-30 10.1093/nar/gkv1265 26582915PMC4702893

# Response

**Published:** 2021-04-30

**Authors:** Francesca Rizzo

**Affiliations:** University of Salerno, Italy

**Q.1** The title should be revised as “a strategy for in-depth analysis of small RNA-seq data” is confusing. The WIND pipeline mainly focuses on the analysis of piRNA, which is only a category of all small RNA species. For example, miRNA is ignored by WIND.

**A.1** The most problematic part of this workflow was, without any doubt, creating a good GTF file and removing all the possible noise from piRNA sequences. We agree that, in this paper, we focused our attention mostly on piRNAs but our workflow is made for analysing all species of sncRNAs as shown in the figures. A user can easily focus his attention on another small RNA species and perform all the analyses exploiting the genomic and the transcriptomic approach. For this reason, we choose to write in the title “piRNAs and beyond”, to indicate that our workflow is suggested not only for the study of piRNAs but can also be used to analyse other sncRNAs molecules.

**Q.2** I agree with the author that the key to piRNA analysis is the comprehensive and accurate identification of piRNAs and piRNA precursor. However, only piRNABank and RNAcentral were used in the “Annotation forging” step of WIND while updated databases of piRNA and piRNA cluster had been published. I suggest the authors integrating piRBase and piRNA cluster database in the WIND pipeline.

**A.2** Thank you for your valuable comment. Initially, we have incorporated piRNAbank in our WIND pipeline as the first building block for the piRNAs annotation track. After that, we incorporated RNACentral to include also sncRNAs sequences in the annotation track in order to have a complete view of the sncRNAs species (known until now).

 However, regarding the additional piRBase database, we are facing a major bottleneck in including it. In detail, the database provides the largest and more comprehensive resource of piRNA sequences annotation, including more than 8 million sequences for human and about 60 million sequences for mouse. A major problem is that many of these sequences are located in the same genomic locus, and they differ only in one or a few nucleotides opening the possibility, not yet demonstrated, that they could be piRNA isoforms produced by variations on 5’ or 3’ end, including nucleotides extension, addition or trimming.

 This situation raises the question of how these molecules should be quantified and including all of them as independent molecules (“In piRBase, if a piRNA sequence is a subsequence of another piRNA, both of them were considered as different sequences and were assigned distinct piRBase names.” cited from Wang *et al.* (2019)) would significantly falsify the abundance ratio in the quantification step when measuring gene expression with Featurecounts or Salmon. Indeed, this could be an additional methodological constraint that would generate biased counts for any further downstream analysis (e.g. differential expression analysis). On this premise, we are considering including piRBase in a future version of WIND, but some issues about the quantification need to be solved. Nevertheless, about piRNAClusterDB (piRNAcldb), as suggested, we integrated the information included in piRNAcldb as metadata in the final GTF file produced by the annotation forging step. We updated the workflow shown in Figure 1 and we added the following sentence in the Method section:

**Q.3** Figure 1. “small RNA Fastq files” were not used in the “Annotation forging” step, and should be placed in the “Pre-processing and quantification” step.

**A.3** We modified the figure as suggested.

**Q.4** In the “Annotation forging” step, I noticed two-length thresholds (100 bp and 69 bp) were used when building the “final Fasta file”. So why 100 and 69?

**A.4** We have selected 100 nts as a filter for sncRNAs sequences on both databases to filter out all other sequences that were too long to be sncRNAs. Following, we added a new filter to the sequences deriving from the piRNABank alignments to the genome (hg38). Although the piRNABank sequences that we used are shorter than 34 nts, when these sequences were aligned to the genome, the genomic ranges were in some cases ≥ 69 nts due to gap-opening, specifically, we found few sequences aligning on genomic regions longer than 33nts (69, 75, 81, 87 and 99 nts). This could be due to the fact that the original piRNABank database was built on genome version hg18. In order to exclude these genomic ranges, which correspond to molecules that do not align correctly on the new version of the genome, we applied the filter at 69 nts as piRNAs are considered to be about 28-34 nts. However, the users can easily change this number or remove this filter as they prefer.

**Minor comments:**

**Q.5** Figure 5 is bar plot rather than histogram.

**A.5** We corrected the error in the figure legend and in the text.

**Q.6** Figure 6B - part of the legend at the bottom is obscured.

**A.6** We corrected the issue.

# Peer review: Reviewer response for version 1

**DOI:** 10.5256/f1000research.30818.r76394

**Published:** 2021-01-14

**Authors:** Juan Pablo Tosar

**Affiliations:** 1 Analytical Biochemistry Unit, School of Science, Universidad de la República, Montevideo, Uruguay; 2 Functional Genomics Unit, Institut Pasteur de Montevideo, Montevideo, Uruguay

The authors developed a workflow for small RNA-seq data analysis, especially intended for the study of Piwi-interacting RNAs or piRNAs. The authors called their workflow WIND (for Workflow for pIrnas aNd beyonD) which is a nice name but should not be presented as an acronym because it is not. The manuscript is professionally written and reads very well, and I think the workflow is complete and can be a useful resource. However, I have major concerns in its design that I will try to explain below.

 One of the motivations of the authors was to develop a reliable package for piRNA analysis that can be also applied for piRNA identification in somatic cells. The authors cite our 2018 study (ref. 25) so they are aware that piRNA databases contain a small percentage of contaminating entries that are probably not piRNAs. They considered this information in the design of their workflow and removed all piRNA reads in piRNABank that also have an alternative annotation in RNA Central. However, it is not clear to me whether they did the same with the piRNA sequences in RNA Central that also have an alternative annotation in RNA Central. Figure 2A shows that roughly 80% of the sequences in testis are piRNAs, and also 80% of the sequences in testis are annotated as tRNAs. So this is the proof that, if the authors really intended to depurate their GTF file from piRNAs also having an annotation in RNA Central, they were not effective in doing so. And this completely alters the author’s conclusions regarding non-germinal piRNAs.

 Another concern is that RNA Central is explicitly a database of non-coding RNAs. Thus, by removing entries in piRNABank that have an alternative annotation in RNA Central, they are not removing those piRNAs in piRNABank that are mRNA-fragments. How to distinguish secondary piRNAs generated from cleavage of coding sequences from mRNA fragments contaminating piRNA databases? It is not surprising, therefore, that the authors found that the remaing piRNAs in their GTF file are either derived from piRNA clusters or from protein-coding genes. This is a methodological bias and does not seem to be a deliberate decision based on the biology of the piRNA pathway. Again, this can completely alter the authors’ results and conclusions when analyzing RNA-seq data obtained in somatic cells using WIND.

 The authors affirm that they focused in “solving on of the most challenging issues of (small RNA) analysis, the annotation controversies of piRNAs”. I’m afraid that, in my opinion, this controversy is still not solved. I would suggest the authors to read our last contribution in this topic (Tosar et al. 2020 ^1^) and reconsider the design of their workflow based on what we discussed in that paper. My suggestion is to take the union of piRNAs from piRNABank and RNA Central, and remove those sequences that have alternative annotations in RNA Central. This can be used to construct GTF file 1 containing “canonical” piRNAs derived from piRNA clusters and also mRNA fragments (whether truly piRNAs or not). Then, remove sequences matching to human or mouse mRNAs from RefSeq to make GTF file 2, containing sequences that can only be classified as piRNAs. Repeat their analysis and compare the results shown in this manuscript with what they see based on my suggested approach.

**Minor comments:**

- Consider adding “testis” and “COLO 205 cells” as a headline in Figure 2.
- The authors refer that the problem of detecting piRNAs in COLO 205 cells is their low expression. However, there are some sequences which are highly expressed according to Figure 2, D. Are these sequences really piRNAs?
- A brief description of the sequencing library preparation should be supplied. If the authors spiked in methylated RNAs, treating the samples with sodium periodate before NGS could have been an interesting control.
- Sequence logos are nice and can be informative, but the workflow could be more powerful if it included plots showing ping-pong signals.
- Why 69 nt as a cut-off?

I hope the authors find this suggestions useful and my comments constructive.

Is the rationale for developing the new method (or application) clearly explained?

Yes

Is the description of the method technically sound?

Yes

Are the conclusions about the method and its performance adequately supported by the findings presented in the article?

Partly

If any results are presented, are all the source data underlying the results available to ensure full reproducibility?

Yes

Are sufficient details provided to allow replication of the method development and its use by others?

Yes

Reviewer Expertise:

Molecular and cell biology

I confirm that I have read this submission and believe that I have an appropriate level of expertise to confirm that it is of an acceptable scientific standard, however I have significant reservations, as outlined above.

## References

[1] : Circulating SNORD57 rather than piR-54265 is a promising biomarker for colorectal cancer: common pitfalls in the study of somatic piRNAs in cancer. *RNA* .2020; 10.1261/rna.078444.120 10.1261/rna.078444.120 33376191PMC7962485

# Response

**Published:** 2021-04-30

**Authors:** Francesca Rizzo

**Affiliations:** University of Salerno, Italy

**Q.1** One of the motivations of the authors was to develop a reliable package for piRNA analysis that can be also applied for piRNA identification in somatic cells. The authors cite our 2018 study (ref. 25) so they are aware that piRNA databases contain a small percentage of contaminating entries that are probably not piRNAs. They considered this information in the design of their workflow and removed all piRNA reads in piRNABank that also have an alternative annotation in RNA Central. However, it is not clear to me whether they did the same with the piRNA sequences in RNA Central that also have an alternative annotation in RNA Central. Figure 2A shows that roughly 80% of the sequences in testis are piRNAs, and also 80% of the sequences in testis are annotated as tRNAs. So this is the proof that, if the authors really intended to depurate their GTF file from piRNAs also having an annotation in RNA Central, they were not effective in doing so. And this completely alters the author’s conclusions regarding non-germinal piRNAs.

**A.1** We thank the reviewer for the helpful suggestion. We removed all piRNA sequences in piRNABank that also have an alternative annotation in RNA Central. However, checking the sequences in RNA Central that also have an alternative annotation in RNA Central, we found only six molecules and none of them is a piRNA. These sequences have been deduplicated in the final GTF, and corresponded to snoRNAs.

 We apologize to the reviewer for the misunderstanding, in Figure 2, the categories on the right of the green dashed line should be referred to the axis on the right. In this specific case, the piRNAs in the testis sample are ~75% while tRNA are ~4% as shown on the axis on the right of the plot. To clarify this point, we specified this in the legend as follows: << The biotypes on the right side of the green dashed line are the least abundant, and the reference values are reported on the Y right axis.>>

**Q.2** Another concern is that RNA Central is explicitly a database of non-coding RNAs. Thus, by removing entries in piRNABank that have an alternative annotation in RNA Central, they are not removing those piRNAs in piRNABank that are mRNA-fragments. How to distinguish secondary piRNAs generated from cleavage of coding sequences from mRNA fragments contaminating piRNA databases? It is not surprising, therefore, that the authors found that the remaining piRNAs in their GTF file are either derived from piRNA clusters or from protein-coding genes. This is a methodological bias and does not seem to be a deliberate decision based on the biology of the piRNA pathway. Again, this can completely alter the authors’ results and conclusions when analyzing RNA-seq data obtained in somatic cells using WIND.

**A.2** We want to thank the reviewer for this challenging question. As suggested in question 3, we have created a new GTF that takes this into account by removing the sequences matching to human or mouse mRNAs. For more details see “answer 3”.

**Q.3** The authors affirm that they focused in “solving one of the most challenging issues of (small RNA) analysis, the annotation controversies of piRNAs”. I’m afraid that, in my opinion, this controversy is still not solved. I would suggest the authors to read our last contribution in this topic (Tosar et al. 20201) and reconsider the design of their workflow based on what we discussed in that paper. My suggestion is to take the union of piRNAs from piRNABank and RNACentral, and remove those sequences that have alternative annotations in RNACentral. This can be used to construct GTF file 1 containing “canonical” piRNAs derived from piRNA clusters and also mRNA fragments (whether truly piRNAs or not). Then, remove sequences matching to human or mouse mRNAs from RefSeq to make GTF file 2, containing sequences that can only be classified as piRNAs. Repeat their analysis and compare the results shown in this manuscript with what they see based on my suggested approach.

**A.3** We want to thank the reviewer for this very illuminating article regarding the piRNA annotation challenges. We agree with the reviewer that the problem has not been completely resolved, but we are trying to move in that direction and above all, we are trying to highlight that this problem must be considered and addressed in order to study the piRNAs correctly. Using the suggestions proposed, we created a new GTF file removing the sequences matching to human or mouse mRNAs. Using this approach, it is possible to note that some differences exist between the previous and the new results. For this reason, we modified all the tables, figures and data in the text accordingly. We are confident that now our workflow is stronger and more robust. However, it is possible to obtain the previous GTF from GitHub or change the code in order to produce the preferred GTF.

 Finally, we have also added on the method section the details about the creation of this new GTF file: <>

 Minor comments:

**Q.4** Consider adding “testis” and “COLO 205 cells” as a headline in Figure 2.

**A.4** We modified the figure as suggested.

**Q.5** The authors refer that the problem of detecting piRNAs in COLO 205 cells is their low expression. However, there are some sequences which are highly expressed according to Figure 2, D. Are these sequences really piRNAs?

**A.5** When we talk about the low expression of piRNAs in COLO205 we are referring to an average expression of all identified molecules, however, among these, there are also molecules with a higher expression. In any case, after the creation of the new GTF file, as described before, we reanalysed all the datasets and we were still able to identify some molecules highly expressed and classified as piRNAs. Obviously, WIND exploits the previous knowledge about sncRNAs, this means that the obtained results, even if more accurate thanks to these new improvements, are still limited due to the primary databases used. However, as always suggested, a wet-lab validation should be necessary to confirm the in silico results and to really establish the correct identification and function of the discovered molecules, but this is beyond the scope of this article.

**Q.6** A brief description of the sequencing library preparation should be supplied. If the authors spiked in methylated RNAs, treating the samples with sodium periodate before NGS could have been an interesting control.

**A.6** As suggested by the reviewer, we added three samples treated with sodium periodate before library preparation and now, in Supplementary Table 2, is available the “Treated” table with the percentages of all the spike-ins used. Furthermore, we modified the method section accordingly:

**Q.7** Sequence logos are nice and can be informative, but the workflow could be more powerful if it included plots showing ping-pong signals.

**A.7** We added in the GitHub repository a code called “pinp_pong.Rmd” that creates a ping-pong plot or a coverage plot for both the strands from a BAM file selected for piRNAs. Finally, we modified the Methods section as following:

**Q.8** Why 69 nt as a cut-off?

**A.8 **We have selected 100 nts as a filter for sncRNAs sequences on both databases to filter out all other sequences that were too long to be sncRNAs. Following, we added a new filter to the sequences deriving from the piRNABank alignments to the genome (hg38). Although the piRNABank sequences that we used are shorter than 34 nts, when these sequences were aligned to the genome, the genomic ranges were in some cases ≥ 69 nts due to gap-opening, specifically, we found few sequences aligning on genomic regions longer than 33nts (69, 75, 81, 87 and 99 nts). This could be due to the fact that the original piRNABank database was built on genome version hg18. In order to exclude these genomic ranges, which correspond to molecules that do not align correctly on the new version of the genome, we applied the filter at 69 nts as piRNAs are considered to be about 28-34 nts. However, the users can easily change this number or remove this filter as they prefer.
